# Screening, prevalence, and risk factors for cervical lesions among HIV positive and HIV negative women in Swaziland

**DOI:** 10.1186/s12889-017-4120-3

**Published:** 2017-02-21

**Authors:** Pauline E. Jolly, Simangele Mthethwa-Hleta, Luz A. Padilla, Jessica Pettis, ShaCoria Winston, Tomi F. Akinyemiju, Hannah J. Turner, Amarachi Ejiawoko, Raina Brooks, Lena Preko, Peter O. Preko

**Affiliations:** 1 Department of Epidemiology, School of Public Health, Ryals Public Health Building, University of Alabama at Birmingham, 1665 University Boulevard Birmingham, Birmingham, AL 35294-0022 USA; 2 Ministry of Health and Social Welfare, 2nd Floor Ministry of Justice & Constitutional Affairs Building, Mhalambanyatsi Road, Mbabane, Swaziland; 3 Swaziland Breast and Cervical Cancer Network (SBCN), Stall A31 Printpak Building, Industrial Site, Sheffield Road, Mbabane, Swaziland; 4 Care and Treatment Lead for the President’s Emergency Plan for AIDS Relief (PEPFAR), Jubela Street, Kent Rock, Mbabane, Swaziland

**Keywords:** Cervical lesions, HIV positive women, VIA, Swaziland

## Abstract

**Background:**

Cervical Cancer (CC) is the number one cancer among women in sub-Saharan Africa. Although CC is preventable, most women in developing countries do not have access to screening.

**Methods:**

This cross-sectional study was conducted to determine the prevalence and risk factors for cervical lesions using visual inspection with acetic acid (VIA) among 112 HIV positive and 161 negative women aged 18–69 years.

**Results:**

The presence of cervical lesions was greater among HIV positive (22.9%) than HIV negative women (5.7%; *p* < 0.0001). In logistic models, the risk of cervical lesions among HIV positive women was 5.24 times higher when adjusted by age (OR 5.24, CI 2.31–11.88), and 4.06 times higher in a full model (OR 4.06, CI 1.61–10.25), than among HIV negative women. In the age-adjusted model women who had ≥2 lifetime sexual partners were 3 times more likely (OR 3.00, CI 1.02–8.85) to have cervical lesions compared to women with one lifetime partner and the odds of cervical lesions among women with a history of STIs were 2.16 greater (OR 2.16, CI 1.04–4.50) than among women with no previous STI. In the fully adjusted model women who had a previous cervical exam were 2.5 times more likely (OR 2.53, CI 1.06–6.05) to have cervical lesions than women who had not.

**Conclusions:**

The high prevalence of HIV infection and the strong association between HIV and cervical lesions highlight the need for substantial scale-up of cervical screening to decrease the rate of CC in Swaziland.

## Background

Cervical cancer (CC) is the fourth most common cancer in women worldwide and the number one cancer among women in sub-Saharan Africa. There were an estimated 528,000 new CC cases worldwide in 2012 and 266,000 CC deaths [1]. About 84% of the global burden of CC occurs in less developed countries with limited resources [1]. Although CC is preventable, most women in resource-poor countries do not have access to a screening program. Gakidou et al. reported that on average CC screening coverage in developing countries is 19% compared to 63% in developed countries; rates as low as 1% or less were seen in countries such as Bangladesh, Ethiopia and Myanmar [2].

CC is primarily caused by the Human Papillomavirus (HPV; [3]). In women with healthy immune systems, most HPV infections are transient and cleared. However, CC is four to five times more prevalent among women who are HIV-positive [4]. Several studies clearly show an increased risk of precancerous cervical lesions and possibly more rapid progression to cancer among HIV positive women. Compared with HIV negative women, HIV positive women are more likely to be infected with HPV and to have persistent HPV leading to pre-cancer, larger and more difficult to treat precancerous lesions, higher recurrence rates of pre-cancer following treatment, and precancerous lesions that progress more rapidly to invasive cancer [5–7]. Invasive CC and its precursor, cervical intraepithelial neoplasia (CIN), are associated with persistent infection with oncogenic ‘high-risk’ (HR) types of HPV [8]. The highest burden of HIV/AIDS is in sub-Saharan Africa and more than half of infected people are women with little or no access to CC screening. Swaziland’s HIV prevalence of 27.4% is the highest in the world [9] and women aged 15–49 years make up more than half of all those infected; HIV prevalence among pregnant women is estimated at 39.2% [10].

An unpublished study titled Female Reproductive Cancer Awareness conducted in Swaziland in 2009 by the Swaziland Breast and Cervical Cancer Network (SBCCN) revealed that CC was the most common cause of cancer-related hospital admissions among Swazi women. This is most likely due to the high prevalence of HIV among women in the country. According to the latest WHO data published in May 2014, CC Deaths in Swaziland reached 112 or 0.83% of total deaths [11]. Cytology-based screening, as used in high-income countries, is currently not feasible in Swaziland because of the financial, infrastructure, human resources and technological investments required to sustain such a program. Visual Inspection with Acetic Acid (VIA) is an evidence-based alternative approach to cytology-based screening for CC in low-resource settings. Studies have reported that the sensitivity of VIA (77%; range 56–94%) for detecting precancerous lesions is comparable to, or greater than, cervical cytology (60%; range 35–84%), while requiring less resources [12]. In addition, VIA provides immediate results, thus promoting linkage of screening with treatment. VIA combined with cryotherapy (freezing of precancerous cervical lesions), ideally in a single visit approach (SVA), is an effective and efficient strategy for secondary prevention of CC in low-resource settings, and can be conducted by competent clinicians and nurses [13]. This linkage of screening with treatment has contributed to VIA-based programs demonstrating a reduction in precancerous lesions, CC incidence, and mortality [12].

In 2010, the SBCCN in partnership with an international donor and the Ministry of Health launched the first three dedicated CC ‘screen and treat’ clinics in the country at the Raleigh Fitkin Memorial Hospital in Manzini, the Mbabane Government Hospital, and the Hlatikulu Government Hospital which has allowed cervical screening for a number of women. Unfortunately, many cases are late-stage diagnosis with very poor prognosis. CC not only significantly increases health care cost, it also places huge financial constraints on the families of affected women due to extensive treatment, supportive care costs, and loss of income. Additionally, it results in significant emotional distress for the families.

Since CC is preventable, identifying potential factors that increase women’s risk for cervical lesions is essential to developing programs that empower women to practice risk-reducing behaviours. Based on the literature, factors such as, younger age at sexual initiation, multiple sexual partners, higher number of recent sexual partners, tobacco use, immune suppression such as caused by HIV infection, high parity, long-term oral contraceptive use and infection with other sexually transmitted infections have been identified as risk factors for CC [8, 14–16]. Further, factors such as knowledge and attitude towards screening, as well as availability and accessibility of services determine the extent to which women will participate in screening and other health services [17].

This cross-sectional study was designed to investigate the prevalence and risk factors for CC among HIV positive women in Swaziland. With a better understanding of the epidemiology of CC in Swaziland health promotion activities that address modifiable risk behaviors and motivate women to take measures to reduce their risk for CC can be developed. Additionally, the results of the study can be used to guide the development of policies and health service delivery related to preventing cervical cancer. The specific objectives of the study were to: 1) estimate the prevalence of cervical lesions/cancer by screening a sample of HIV positive and HIV negative women for cervical lesions/cancer using VIA; 2) Assess the association between socio-demographic factors and CC among the women; and 3) determine the association between sexual and reproductive history and CC among the women.

## Methods

### Study design and study site

A cross-sectional study was conducted among HIV positive and HIV negative women aged 18–69 years. The women were recruited from three hospitals in three regions of Swaziland that provided VIA and cryotherapy, namely, the Mbabane Hospital in the Hhohho Region, the Raliegh Fitkin Memorial (RFM) Hospital in the Manzini Region, and the Hlatikulu Hospital in the Shiselweni Region. These are all regional referral hospitals although Mbabane is a national referral hospital. These were the only facilities with VIA and cryotherapy services at the time of the study although such services have since been scaled up. HIV patients were selected from antiretroviral therapy (ART) clinics and from among patients who tested HIV positive at HIV care and treatment (HTC) sites at the out-patient departments (OPDs) of the hospitals. The comparison group were women who tested HIV negative at the OPDs.

### Recruitment and data collection

Potential participants were informed of the study by the clinic staff and those who expressed interest in participating were introduced to the study staff by the clinic staff. The study staff then told the women about the purpose and procedures of the study. The women were informed that participation was voluntary and that they could refuse to participate and could withdraw from the study at any time. Women 18–69 years who lived in the specific region and did not have cervical complaints were eligible to participate in the study; cases had to be HIV-positive and controls had to be HIV-negative. Pregnant women and women who were less than 12 weeks postpartum were excluded until they were greater than 12 weeks postpartum. Women who expressed interest in the study were asked to read the informed consent and to ask questions before signing the form. After providing informed consent, a questionnaire was administered by trained interviewers to collect data on:1) Socio-demographic and lifestyle characteristics (age, sex, marital status, income, education, occupation, smoking, substance and alcohol abuse); and 2) Sexual and reproductive practices (condom use, multiple partners, age of sexual initiation, age at first child). A unique participant ID number was placed on each questionnaire instead of personal identifying information. The interviews were conducted in private hospital rooms to ensure confidentiality and took approximately 45 min to complete. After the interview each woman was screened for cervical lesions by a trained clinic staff member using VIA. A woman was considered VIA positive if there was a thick, dense white lesion with distinct borders located within the transformation zone of the cervix and close to or touching the squamocolumnar junction (SCJ; [18]). The study protocol was reviewed and approved by the Institutional Review Board of the University of Alabama at Birmingham and the Scientific and Ethics Committee of the Ministry of Health and Social Welfare, Kingdom of Swaziland, prior to implementation.

### Data analysis

Data were analyzed for 273 women (112 HIV-positive and 161 HIV-negative) aged 18–69 years.

VIA results were available for 271 of the women but HIV status is not available for three. Socio-demographic and sexual and reproductive history variables were tabulated for HIV positive and negative and VIA positive and negative women. Descriptive statistics (frequencies, percentages, means) were performed on all variables and used to summarize the socio-demographic and sexual and reproductive history data for the study groups. Differences among the variables for the groups were compared using Chi-square and *t*-test analyses. Variables that were associated with cervical lesions and other relevant variables were used to create multivariate models. Logistic regression was used to estimate odds ratios and 95% confidence intervals for the risk of cervical lesions based on VIA results and other significant sexual history variables, adjusting for socio-demographic variables. SAS 9.4 (SAS institute, Cary, NC) software was used for the analysis and all statistical tests of a two-sided *p*-value of <0.05 were considered significant.

## Results

When bivariate analysis was conducted between sociodemographic variables and HIV status, significant associations were found between HIV status and age, income, and level of education (Table 1). A higher proportion of HIV positive women were ≥age 30 years compared with HIV negative women (*p* = 0.04). On the contrary, a higher proportion of HIV negative women earned higher income (>E1201) per month (*p* = 0.03) and had a higher level of education than HIV positive women (*p* <0.01). With regard to cervical lesions, four times as many HIV positive women (22.9%) compared with HIV negative women (5.7%) had cervical lesions (*p* <0.01) based on VIA results. When bivariate analysis was conducted between sociodemographic and sexual and reproductive history variables and VIA status none of the sociodemographic variables was significantly associated with presence of cervical lesions (Table 2). However, there were significant associations between the number of lifetime sexual partners and the presence of cervical lesions (*p* = 0.03), and between VIA and HIV status (Table 2). A significantly higher proportion of VIA positive women (73.5%) were HIV positive compared with HIV negative women (35.96%; *p* <0.01). A marginal association was observed between a history of STI and the presence of cervical lesions (*p* = 0.05). Although cervical lesions did not differ according to age at first sexual intercourse or age at first child, a substantial portion of the women (40.6%) had their first sexual intercourse at or below age 17 years (48.6% of women with cervical lesions and 39.4% of women without). The age at first child was at or below 17 years for 35.8% of the women.

**Table 1:** Sociodemographic variables by HIV status and visual inspection by acetic acid (VIA) results

	HIV positive *N* = 112	HIV negative *N* = 161	*p*-value	VIA positive *N* = 35	VIA negative *N* = 236	*p*-value
	*N* (%)	*N* (%)		*N* (%)	*N* (%)	
Age						
<30	32 (28.6)	65 (40.4)	**0.04**	11 (31.4)	75 (31.8)	0.97
≥30	80 (71.4)	94 (59.6)		24 (68.6)	161 (68.2)	
Region						
Hhohho	41 (36.6)	67 (42.4)	0.77	18 (51.4)	92 (39.5)	0.11
Lubombo	3 (2.7)	4 (2.5)		2 (5.7)	5 (2.1)	
Manzini	56 (50.0)	69 (43.7)		14 (40.0)	106 (45.5)	
Shiselwenni	12 (10.7)	18 (11.4)		1 (2.9)	30 (12.9)	
Had previous cervical exam						
Yes	34 (30.6)	48 (30.2)	0.94	15 (42.9)	65 (27.9)	0.08
No	77 (69.4)	111 (69.8)		20 (57.1)	168 (72.1)	
Marital Status						
Married/Cohabiting	48 (42.9)	74 (46)	0.61	15 (42.8)	106 (44.9)	0.82
Widowed/Single/Separated	64 (57.1)	87 (54)		20 (57.1)	130 (55.1)	
Monthly income (1US = 10E)						
<E500 or None	38 (33.9)	61 (38.1)	**0.03**	11 (31.4)	88 (37.5)	0.43
E500–1200	38 (33.9)	32 (20)		12 (34.3)	56 (23.8)	
>E1201	36 (32.1)	67 (41.9)		12 (34.3)	91 (38.7)	
Highest educational level						
Primary/No school	33 (29.5)	25 (15.5)	**<0.01**	6 (17.1)	50 (21.2)	0.08
Secondary	70 (62.5)	90 (55.9)		26 (74.3)	134 (56.8)	
Tertiary	9 (8)	46 (28.6)		3 (8.6)	52 (22.0)	
Employment status						
Employed/Self-employed/Partially	75 (67)	93 (59.2)	0.20	26 (74.3)	140 (60.3)	0.12
Unemployed/Student	37 (33)	64 (40.8)		9 (25.7)	92 (39.7)	
Occupation						
Laborer	31 (38.7)	35 (32.7)	0.68	12 (44.4)	52 (32.7)	0.13
Skilled worker/Clerical	29 (36.3)	44 (41.1)		6 (22.2)	67 (42.1)	
Professional	20 (25)	28 (26.2)		9 (33.3)	40 (25.2)	
Religion						
Christian	109 (97.3)	157 (98.7)	0.40	35 (100)	229 (97.9)	1.00
Other (Traditional, Muslim, None)	3 (2.7)	2 (1.3)		0 (0)	5 (2.1)	
Smoking status						
Never smoked	106 (94.6)	157 (97.5)	0.22	35 (100)	226 (95.8)	0.37
Former/Current smoker	6 (5.4)	4 (2.5)		0 (0)	10 (4.2)	
Drink alcohol?						
Yes	12 (10.9)	11 (6.9)	0.25	4 (11.8)	19 (8.2)	0.51
No	98 (89.1)	148 (93.1)		30 (88.2)	214 (91.8)	

Numbers may not always add up to total number due to missing responses. VIA data were available for 271 women but HIV status was not available for 3 of these women

Significant at p<0.05 are in bold

**Table 2:** Sexual and reproductive history according to presence/absence of cervical lesions by visual inspection with acetic acid (VIA)

Variables	VIA Positive *N* = 35	VIA Negative *N* = 236	Total *N* = 271	*p*-value
	*N* (%)	*N* (%)	*N* (%)	
Age at first sexual intercourse				
≤17	17 (48.6)	93 (39.4)	110 (40.6)	0.49
18–23	17 (48.6)	129 (54.7)	146 (53.9)	
≥24	1 (2.9)	14 (5.9)	15 (5.5)	
Number of lifetime sexual partners				
1	4 (11.4)	66 (28.0)	70 (25.8)	**0.03**
≥2	31 (88.6)	170 (72.0)	201 (74.2)	
Mean number of children	2 ± 1.44	2.1 ± 1.90	2.1 ± 1.84	0.64
Age at first child				
≤17	13 (37.1)	84 (35.6)	97 (35.8)	0.36
18–23	20 (57.1)	120 (50.8)	140 (51.7)	
≥24	2 (5.7)	32 (13.6)	34 (12.5)	
Condom use				
Do not use condoms	5 (14.3)	50 (21.2)	55 (20.3)	0.40
Sometimes/Most times/Non-Regular partner only	23 (65.7)	127 (53.8)	150 (55.3)	
100% of the time	7 (20.0)	59 (25.0)	66 (24.4)	
Condom use at last sex				
Yes	17 (48.6)	128 (54.5)	145 (53.7)	0.52
No	18 (51.5)	107 (45.5)	125 (46.3)	
Ever been raped				
Yes	1 (2.9)	15 (6.4)	16 (5.9)	0.70
No	34 (97.1)	221 (93.6)	255 (94.1)	
Sex in exchange for money				
Yes	1 (2.9)	3 (1.3)	4 (1.5)	0.43
No	34 (97.1)	233 (98.7)	267 (98.5)	
Sex in exchange for food or housing/rent?				
Yes	1 (2.9)	3 (1.3)	4 (1.5)	0.43
No	34 (97.1)	233 (98.7)	267 (98.5)	
History of any of the following STIs				
Warts, gonorrhea, syphilis, herpes	15 (42.9)	60 (26.2)	75 (28.8)	0.05
None	20 (57.1)	169 (73.8)	189 (71.6)	
Anal sex in the past 12 months				
Yes	2 (5.7)	11 (4.7)	13 (4.8)	0.68
HIV Status				
Positive	25 (73.5)	84 (34.9)	109 (40.7)	**<0.01**
Negative	9 (26.5)	150 (64.1)	159 (59.3)	

Numbers may not always add up to total number due to missing responses

Significant at p<0.05 are in bold

Table 3 shows the results of age adjusted and fully adjusted logistic models for sociodemographic and sexual and reproductive history variables listed in Tables 1 and 2 and cervical lesions. In both models, HIV was found to be a significant predictor for having cervical lesions. The risk of cervical lesions among HIV positive women was 5 times greater in the age-adjusted model (OR 5.24, CI 2.31–11.88) and 4 times greater in the full model (OR 4.06, CI 1.61–10.25) than among HIV negative women. In the age-adjusted model, number of lifetime sexual partners and history of STI were significant predictors of having cervical lesions. Women who had two or more lifetime sexual partners were 3 times more likely to have cervical lesions (OR 3.00, CI1.02–8.85) compared to women with one lifetime partner. Women who had been diagnosed with a STI were 2 times more likely to have cervical lesions (OR 2.16, CI 1.04–4.50) than women who had not previously been diagnosed with a STI. In the fully adjusted model, previous cervical exam was found to be significantly associated with the presence of cervical lesions. Women who had had a previous cervical exam were 2.5 times more likely to have cervical lesions (OR 2.53, CI 1.06–6.05) than women who had not had a cervical exam.

**Table 3:** Multivariable association of sociodemographic characteristics and sexual and reproductive history with presence of cervical lesions

Variables	Age-adjusted OR (95% confidence interval)^a^	Fully adjusted OR (95% confidence interval)^b^
HIV status		
Positive	**5.24 (2.31–11.88)**	**4.06 (1.61–10.25)**
Negative	Referent	Referent
Age		
<30		2.33 (0.88–6.10)
≥30		Referent
Had previous cervical exam		
Yes	2.09 (0.99–4.41)	**2.53 (1.06–6.05)**
No	Referent	Referent
Marital status		
Married/Cohabiting	Referent	Referent
Widowed/Single	1.01 (0.48–2.09)	0.83 (0.37–1.90)
Monthly income (1US = 10E)		
<E500 or None	Referent	Referent
E500–1200	1.77 (0.72–4.33)	0.78 (0.19–3.14)
>E1201	1.11 (0.46–2.68)	0.75 (0.19–2.93)
Highest educational level		
Primary/No School	Referent	Referent
Secondary	1.50 (0.57–3.93)	2.47 (0.84–7.31)
Tertiary	0.45 (0.11–1.91)	1.11 (0.20–6.34)
Employment status		
Employed/Self/Partially	2.33 (0.99–5.47)	3.12 (0.81–12.11)
Unemployed/Student	Referent	Referent
Age at first sexual intercourse		
≤17	2.30 (0.28–18.81)	0.85 (0.08–9.65)
18–23	1.65 (0.20–13.44)	0.69 (0.06–7.50)
≥24	Referent	Referent
Number of lifetime sexual partners		
1	Referent	Referent
≥2	**3.00 (1.02–8.85)**	2.42 (0.65–9.08)
History of any of the following STIs		
Warts, Gonorrhea, Syphilis, Herpes	**2.16 (1.04–4.50)**	1.42 (0.63–3.22)
No	Referent	Referent
Condom use		
Not used	Referent	Referent
Sometimes/most times/Non-regular partner only	1.61 (0.56–4.64)	1.11 (0.34–3.64)
100% of the time	1.11 (0.33–3.78)	0.76 (0.20–2.96)

^a^Odds Ratio (OR) for each socio-demographic and reproductive variable adjusted for age

^b^Fully adjusted model including all socio-demographic and reproductive variables

The boldface odds ratios are significant

## Discussion

HIV was found to be a significant predictor for presence of cervical lesions in both the age-adjusted and the fully-adjusted logistic models. This is not a surprising finding since HPV, especially high-risk (hr) HPV types, have been found at higher rates in immunosuppressed HIV infected women [6, 7, 19, 20]. Although some of the women in our study were recruited from ART clinics, we did not collect data on time and adherence to ART, or on CD4 count, so we are not able to relate cervical lesions to immune status of the women. The rates of cervical lesions obtained in our study are similar to those found in studies conducted by Yamada et al. in Kenya that reported 21.0% low grade squamous intraepithelial lesions (LSIL) in HIV positive women versus 6.9% in HIV negative women [20], and by Leroy et al. in Rwanda that reported SILs of 24.3% in HIV positive and 6.5% in HIV negative women [21]. The overall percent of cervical lesions (12.7%) obtained in our study is also similar to the 11.7% cervical dysplasia rate reported by La Ruche et al. among women in the Ivory Coast [22].

With regard to risk factors associated with cervical lesions we found in our age-adjusted model that the number of lifetime sexual partners and a history of STI(s) were significant predictors of cervical lesions. Approximately 74% of the women reported two or more lifetime sexual partners with significantly more HIV positive women (88.6%) reporting two or more lifetime sexual partners compared to HIV negative women (72.0%). Also, STIs occurred fairly regularly among the women with 28.8% reporting a history of STIs. A significantly higher proportion of VIA positive women (42.9%) reported history of STIs compared to VIA negative women (26.2%). Other studies have reported number of sexual partners [16, 23] and STIs [22] as predictors of cervical lesions. Condom use was low or irregular in this population with 20.3% of women reporting no condom use and only 24.4% reporting consistent condom use; the majority of women (55.3%) reported inconsistent condom use. Condom use would be beneficial in protecting against STIs although it is not fully protective against HPV infection which can be transmitted by skin to skin contact.

In the fully adjusted model, previous cervical exam was found to be significantly associated with the presence of cervical lesions. This finding may relate to the fact that women with cervical lesions were more likely to report previous STI(s) or were more likely to be HIV positive and to have received cervical examination. No effort was made in the study to identify or recruit women with prior abnormal lesions. The women were invited to participate in the study as they came into the clinic and only those who agreed and gave informed consent were enrolled. Therefore, there was no recruitment bias according to presence or absence of cervical lesion. Rather, there may be self-selection bias from women who self-selected to participate in the study, were more exposed to the health care system, and had prior cervical exams. Women who newer to the clinic may have been less likely to participate. This finding of association of previous cervical exam with the presence of cervical lesions may also be explained by other unaccounted factors.

This study has other limitations which must be considered in interpreting the results. First it was a cross-sectional study and so does not allow for determination of temporal relationships. Secondly, no ART or CD4 data were collected, and no HPV genotyping was done. These data would have helped in interpreting the results and in further analyses. VIA lesions were not categorized into low-grade SIL or high grade SIL which limits comparison with other published papers. However, this is the first study to estimate cervical lesions using a low resource but highly effective method of screening for cervical lesions in Swaziland.

## Conclusion

The findings that number of sexual partners and history of STI are predictors of cervical lesions can be used to develop and conduct interventions to decrease number of sex partners and protect against STIs among the women. The high prevalence of HIV infection in Swaziland and the strong association between HIV and HPV infections and cervical lesions, highlight the need for substantial scale-up of cervical screening, not only by VIA but also by HPV genotyping, to decrease the high rate of CC in the country.

## References

[1] GLOBOSCAN Cancer Fact Sheets: Cervical cancer. Cervical Cancer Estimated Incidence, Mortality and Prevalence Worldwide in 2012. http://globocan.iarc.fr/old/FactSheets/cancers/cervix-new.asp. Accessed 2 Feb 2017.

[2] Gakidou E, Nordhagen S, Obermeyer Z. Coverage of cervical cancer screening in 57 countries: low average levels and large inequalities. PLoS Med. 2008;5(6):e132. http://journals.plos.org/plosmedicine/article/file?id=10.1371/journal.pmed.0050132&type=printable. Accessed 13 Feb 2017. 10.1371/journal.pmed.0050132 PMC2429949 18563963

[3] Division of STD Prevention, Centers for Disease Control and Prevention. Prevention of genital HPV infection and sequelae: report of an external consultants’ meeting, 1999. http://www.cdc.gov/std/hpv/HPVSupplement99.pdf. Accessed 23 Jan 2017.

[4] Odendal L. CC in women with HIV. AIDSMAP; 2011. http://www.aidsmap.com/Cervical-cancer-in-women-with-HIV/page/1669155/. Accessed 23 Jan 2017.

[5] Branca M, Gabruglia AR, Benedetto A (2003). Factors predicting the persistence of genital human papillomavirus infections and pap smear abnormality in HIV-positive and HIV-negative women during prospective follow up. Int J STD AIDS.

[6] De Vuyst H, Lillo F, Broutet N, Smith JS (2008). HIV, human papillomavirus, and cervical neoplasia and cancer in the era of highly active antiretroviral therapy. Eur J Cancer Prev.

[7] Parham GP, Sahasrabuddhe VV, Mwanahamuntu MH (2006). Prevalence and predictors of squamous intraepithelial lesions of the cervix in HIV-infected women in Lusaka, Zambia. Gynecol Oncol.

[8] National Institutes of Health (NIH) and National Cancer Institute (NCI). HPV and Cancer, 2015. http://www.cancer.gov/about-cancer/causes-prevention/risk/infectious-agents/hpv-fact-sheet. Accessed 23 Jan 2017.

[9] AVERT. HIV and AIDS in Swaziland: Global Report, 2013. http://www.avert.org/professionals/hiv-around-world/sub-saharan-africa/swaziland. Accessed 23 Jan 2017.

[10] United Nations International Children’s Emergency Fund (UNICEF). Swaziland: HIV and AIDS, 2013. http://www.unicef.org/swaziland/hiv_aids.html. Accessed 23 Jan 2017.

[11] World Health Rankings. Top 20 causes of death Swaziland: Cervical Cancer, 2013. http://www.worldlifeexpectancy.com/swaziland-cervical-cancer. Accessed 23 Jan 2017.

[12] Alliance for Cervical Cancer Prevention (ACCP). Cervical Cancer Prevention FACT SHEET: New evidence on the impact of cervical cancer screening and treatment using HPV DNA tests, visual inspection, or cytology, 2009. http://www.rho.org/files/ACCP_screening_factsheet_July09.pdf. Accessed 23 Jan 2017.

[13] Sherris J, Wittet S, Kleine A, Sellors J, Luciani S, Sankaranarayanan R, Barone MA (2009). Evidence-based, alternative cervical cancer screening approaches in low-resource settings. Int Perspect Sex Reprod Health.

[14] World Health Organization Media Centre. Human papillomavirus (HPV) and cervical cancer. Fact sheet N°380, 2015. http://www.who.int/mediacentre/factsheets/fs380/en/. Accessed 23 Jan 2017.

[15] Moscicki AB (2001). Risks for incident human papillomavirus infection and low-grade squamous intraepithelial lesion development in young females. JAMA.

[16] Shi R, Devarakonda S, Liu L, Taylor H, Mills G (2014). Factors associated with genital human papillomavirus infection among adult females in the United States, NHANES 2007–2010. BMC Res Notes.

[17] Coronado-Interis E, Anakwenze CP, Aung M, Jolly PE (2015). Increasing cervical cancer awareness in Jamaica: effectiveness of a theory-based educational intervention. Int J Environ Res Public Health.

[18] Alliance for Cervical Cancer Prevention 2007 Cervical Cancer Prevention FACT SHEET: 10. Key Findings and Recommendations for Effective Cervical Cancer Screening and Treatment Programs*,* ACCP, Seattle, WA. Available at: http://screening.iarc.fr/doc/ACCP_recs_2007_factsheet_final.pdf. Accessed 13 Feb 2017.

[19] Sahasrabuddhe VV, Mwanahamuntu MH, Vermund SH (2007). Prevalence and distribution of HPV genotypes among HIV-infected women in Zambia. Brit J Cancer.

[20] Yamada R, Sasagawa T, Kirumbi LW (2008). Human papillomavirus infection and cervical abnormalities in Nairobi, Kenya, an area with a high prevalence of human immunodeficiency virus infection. J Med Vir.

[21] Leroy V, Ladner J, De Clercq A, Meheus A, Nyiraziraje M, Karita E, Dabis F (1999). Cervical dysplasia and HIV type 1 infection in African pregnant women: a cross sectional study, Kigali, Rwanda. Sex Transm Infect.

[22] La Ruche G, Ramon R, Mensah-Ado I (1998). Squamous intraepithelial lesions of the cervix, invasive cervical carcinoma, and immunosuppression induced by human immunodeficiency virus in Africa. Cancer.

[23] Ursu RG, Onofriescu M, Luca A, Prisecariu LJ, Salceanu SO, Nemescu D, Iancu LS (2015). The need for cervical cancer control in HIV-positive and HIV-negative women from Romania by primary prevention and by early detection using clinically validated HPV/DNA tests. PLoS One.

